# Supplementary material for: Chromothripsis during telomere crisis is independent of NHEJ, and consistent with a replicative origin
Source: Genome Res. 2019 May;29(5):737–49. doi: 10.1101/gr.240705.118 (PMC6499312; doi:10.1101/gr.240705.118)
Supplement: Supplemental Material [file supp_gr.240705.118_Supplemental_file_1.zip › contigs/annotated_contigs/DB57/contig.2.DB57_length_590_mean_cov_3.20169491525.docx]

**DB57_length_590_mean_cov_3.20169491525**

```
TAGAGGAGTTCCAATTCCTAGGATTGTGGAGCGAAAGCGAGAAAGGAGTTTACTCCAAATTGCTCATTTTATCAATAAGGAAGCTGCGG
 >chr17:73112806-73113220 + E=2e-231 p=4e-02
TGCAGACAGCTTCCCCTCGTCTGGCTGTCGCAGGCCAGGATGAGAATGCAGGCTTCCTTCCGTCTGTCCCTGCAGCCACCACACTCACA

GGTACCCTTCCATGCTGGCTTTGCTGTGGCAGCTCTGAGTGAGGTCTGCTCTTTGGCCCAGCACTTCTGCCTCCTCTCTGAGGGCTGGA

AGCTGGATGCTATCCAAACAAATGGATTCGCTCACCTTGCACACTAAGGCCAATTACTGACAAAAATCGCAGTGAGAAAGTGAGGCTTT

GTTTTTGTTTTTGGTTTTGGTTTTTTTGAGACAGATTCTCACTCCGTTGCCCAGGT|TG|ATGCTTCTGACAAGTGCTTAGCTGGCGCC
                                                         >chr17:73115819-73115997 - E=2
TGGCACCTGGTGGCTGGGCAGGCCCTCGGCAAGGGCAGCTCAAAATACAGCTACATCTGGGGACTCGCCCATGCTGAGCTGAAGCAGCC
e-80
CAGGGTGACAGTCTTCCCAGCAGGCTTGGCGTGGTGGCTCACGCCTGTAATCCTAGCA
```
